# Progress of Surgery

**Published:** 1901-05-11

**Authors:** 


### PROGRESS OF SURGERY.

OTOLOGY (*concluded*).

**Mastoiditis.**—Henry A. Alderton (Brooklyn)[1] records an interesting case of mastoid abscess with complications. The patient, a man of twenty-five, had had chronic ear suppuration for twenty-three years. Five weeks before Alderton saw him, he began to have pain over the left mastoid, and fever coincident with cessation of ear discharge. A general surgeon had then operated. A month later, and four days before a final operation, a swelling appeared in front of and above the ear. Two days later there was a rigor with intense headache and a swelling formed under the left eye. Pus then escaped from the wound and the pain became easier, but the swelling continued to spread over the lower jaw up to the level of the pre-molar teeth. The swelling in the end extended backwards from the outer angle of the eye and involved the whole mastoid process. A probe passed through the old operation wound an inch behind the meatus discovered necrosed bone above the auricle. The meatus was swollen and full of cholesteatomatous material, pus, and granulation tissue. A free incision was made over the mastoid and continued forwards over the auricle. Fœtid pus escaped from beneath the periosteum through the horizontal part of the incision. Similar pus was found in the antrum after chiselling through the sclerosed mastoid process. The attic and aditus were carious, and the probe passed through the tegmen antri into the cranial cavity. The carious parts were curetted. The outer table of the skull was found necrotic for a square inch situated 1½ inches above the meatus. Fœtid pus oozed through this area. The inner table was exposed and found to be sequestrous over a much larger area, and was removed in pieces. The dura was adherent to the bone around this area, shutting off the abscess, but a probe passed along a fistulous channel leading down through the tegmen into the antrum. The bridge of bone lying over this fistulous channel was removed. The dura was covered with granulations. The middle ear was curetted. The patient made a good recovery. Eight months later the operation wound was still in connection with the middle ear, but both the wound and middle-ear tract were firmly epidermised and without secretion. James K. Love[2] (Glasgow) has written on the evolution of the mastoid operation. Credit is given to Schwartze for the present position of the radical mastoid operation. The weakness of the Schwartze operation, *i.e.*, the operation of laying open into one smooth-walled cavity all the bony recesses in the tympano-mastoid area, is the double channel left for drainage, viz., that of the external auditory canal and of the mastoid wound. These channels meet at their inner ends, and it is at this place that the hitch is apt to occur which necessitates a second operation on the mastoid after an interval of months or years. The cause of the failure is the presence between the two branches of the healing sinus of a partition—the posterior cutaneo-cartilaginous wall of the external auditory canal. The slitting of this at the time the mastoid operation is performed allows of the immediate closure of the skin wound behind, and the performance of all after-treatment by a single channel—the slit external auditory canal. If this after-treatment be carefully conducted and the wound made to heal from the bottom, free access to the deepest parts of the bony recesses will always be got, and final healing without a second mastoid operation. The advantages claimed for this operation are:—(1) The mastoid wound heals quickly, and there is left no pit or even visible cicatrix; (2) all dressings can be removed from the side of the head in about a fortnight, and the patient may thereafter resume work; (3) a complete control is maintained over the healing of the deep parts, and ultimate cessation of discharge is assured. Skin grafting by Ballance's method, while giving good results, is considered unnecessary in ordinary cases, as healing will take place rapidly enough without it if the canal be carefully packed every two or three days. Richard Lalke[3] contributes a note on the question of retaining the skin of the posterior meatal wall in the radical mastoid operation, and supports the suggestion of Moll, of Arnheim, to remove entirely the posterior portion of the meatus, but not for the reasons, urged by Moll, that the procedure facilitated healing. Lakle has found that very frequently, two years or so after the operation, the patients return with deafness, and the cause is that the whole post-operation cavity has become filled with a mass of cerumen. The explanation is that the ceruminous glands, which lie almost entirely in the posterior meatal wall, have been transplanted to a position from which the exuded cerumen is unable to escape. The delayed appearance of the deafness is due partly to the large cavity there is to fill, and partly to the suspension of glandular activity for some period subsequent to the operation. Albert H. Andrews[4] has described a new test for mastoiditis. A small-belled stethoscope is placed on the tip of the mastoid, while the handle of a vibrating tuning-fork is held over the mastoid antrum. When the mastoid is filled with pus or granulations the sound is transmitted to the ears of the examiner more distinctly than on the normal side. In normal cases there is no pereptible difference between the two sides. A case is reported in which a cholesteatomatous mass filled the attic and upper part of the middle ear. The tuning-fork was heard on this side for thirty seconds, while on the normal side it was heard only sixteen seconds. He uses a C 512 tuning-fork which vibrates about thirty-five seconds, and a stethoscope with a ⅝-inch bell.

**Atresia Auris Congenita.**—Hunter Tod[5] has contributed a very complete account of this condition and tabulated a large number of cases. The literatures he has found to be abundant and complete, thanks chiefly to German writers. Tod records three cases he has himself lately seen. He has collected 114 cases from literature. Of these he gives particulars of 57 only, because Ruedi had previously considered the other 57 cases independently. One of Tod's cases may be briefly described, that of a man of 25. There was congenital bilateral aural deformity, with total atresia of both meatus. Careful operation twelve years before had been unsuccessful. Hearing was sufficiently good to carry on conversation and daily occupation. Tuning-forks were heard well in the upper half of the scale, but not below $C_1$, and there was marked increase of bone conduction for lower tones. No other deformity present. The Eustachian tubes were patent. Bougies could be passed nearly two inches. Hearing was better with the mouth open. The chief complaint was tinnitus. Paracusis Willisii was well marked. We have only further space to quote Tod's summarised conclusions concerning this affection. These are:—1. The deformity is not hereditary and the cause is not known. 2. It is more often unilateral than bilateral. 3. There may be accompanying deformities, chiefly due to mal-development of the parts in connection with the first and second branchial arches. 4. The labyrinth is rarely affected. The hearing varies, but is present to some extent, though slightly. Hearing tests give practically the same results as those in uncomplicated middle-ear affections, but more marked. 5. Embryological, pathological, and clinical observations prove operation to be useless. 6. Something more may be done, perhaps, by careful non-operative treatment, and by early and assiduous instruction in speaking and lip-reading.

[1] Brooklyn Med. Jour., Nov. 1900. [2] Glasgow Med. Jour., Dec. 1900. [3] Jour. Laryng. R. and O., March, 1901. [4] Jour. Amer. Med. Assoc., Jan. 1901. [5] Jour. Laryng. R. and O., March, 1901.

